# Inconspicuous Yet Indispensable: The Coronavirus Spike Transmembrane Domain

**DOI:** 10.3390/ijms242216421

**Published:** 2023-11-16

**Authors:** Elena T. Aliper, Roman G. Efremov

**Affiliations:** 1 Shemyakin-Ovchinnikov Institute of Bioorganic Chemistry, Russian Academy of Sciences, Moscow 117997, Russia; 2 Department of Applied Mathematics, National Research University Higher School of Economics, Moscow 101000, Russia; 3 L.D. Landau School of Physics, Moscow Institute of Physics and Technology (State University), Dolgoprudny 141701, Russia

**Keywords:** viral fusion protein, transmembrane helical trimer, protein–protein interactions in membrane, membrane protein structure, molecular modelling, viral protein acylation, helix–helix interface

## Abstract

Membrane-spanning portions of proteins’ polypeptide chains are commonly known as their transmembrane domains (TMDs). The structural organisation and dynamic behaviour of TMDs from proteins of various families, be that receptors, ion channels, enzymes etc., have been under scrutiny on the part of the scientific community for the last few decades. The reason for such attention is that, apart from their obvious role as an “anchor” in ensuring the correct orientation of the protein’s extra-membrane domains (in most cases functionally important), TMDs often actively and directly contribute to the operation of “the protein machine”. They are capable of transmitting signals across the membrane, interacting with adjacent TMDs and membrane-proximal domains, as well as with various ligands, etc. Structural data on TMD arrangement are still fragmentary at best due to their complex molecular organisation as, most commonly, dynamic oligomers, as well as due to the challenges related to experimental studies thereof. Inter alia, this is especially true for viral fusion proteins, which have been the focus of numerous studies for quite some time, but have provoked unprecedented interest in view of the SARS-CoV-2 pandemic. However, despite numerous structure-centred studies of the spike (S) protein effectuating target cell entry in coronaviruses, structural data on the TMD as part of the entire spike protein are still incomplete, whereas this segment is known to be crucial to the spike’s fusogenic activity. Therefore, in attempting to bring together currently available data on the structure and dynamics of spike proteins’ TMDs, the present review aims to tackle a highly pertinent task and contribute to a better understanding of the molecular mechanisms underlying virus-mediated fusion, also offering a rationale for the design of novel efficacious methods for the treatment of infectious diseases caused by SARS-CoV-2 and related viruses.

## 1. Introduction

The SARS-CoV-2 pandemic of 2020–2022 became a poignant revelation of how important it is to possess detailed information on the structural organisation of essential viral proteins, most notably of the spike protein, which is responsible for effective receptor recognition on the target cell surface, binding thereto and subsequent membrane fusion. These data are required in order to understand the molecular mechanisms of the virus’s functioning at the initial stages of infection and, consequently, to effectuate the rational design of antiviral agents. It thus comes as no surprise that the situation around COVID-19 triggered an exponential growth of structure-based studies dedicated not only to the spike protein, but also to its complexes with receptors or antibodies (reviewed in, e.g., [1,2]). In such studies, chief attention has been paid to the behaviour of the receptor-binding domain (RBD) as the one most heavily involved at the initial stages of infection [1,3,4] and, to a lesser extent, to the N-terminal domain. Meanwhile, the role of the transmembrane domain (TMD) and a number of other portions of the spike protein engaged in interaction with the virus’s own envelope and target cell membrane have been studied much less thoroughly. One exception would clearly be the fusion peptides active at the initial stages of the fusion machinery operation (see [5] for a detailed account), whereas on a number of occasions authors of structure-based studies on the spike protein only consider the TMD as a physical anchor required to firmly fix the protein in the viral envelope and not affecting the interaction with the receptors on the target cell surface.

However, knowledge on the structure and dynamics of integral membrane proteins accumulated over the years, especially recently, unambiguously demonstrates that a TMD acting as nothing but an anchor is an exception to the rule (e.g., [6,7]). In many cases it is none other than the TMD that plays a key role in the functioning of such proteins, alongside membrane-proximal regions and domains that do not contact the membrane initially, but interact therewith as the protein goes on to perform its function. This scenario implies that, on the one hand, the TMD must possess a rigid structure to act as a mechanical “support” for the extra-membrane domains, which are often large and highly mobile, and, on the other hand, it must contribute towards all the finely regulated conformational rearrangements, smoothly guiding the entire system through all the required intermediate states. As an example, one can evoke cellular signalling effectuated, inter alia, by receptor tyrosine kinases (reviewed in [8]), in the case whereof TMD oligomerisation as well as the specificity and dynamics of protein/membrane interactions predetermine the parameters of receptor activation. Notably, such phenomena are only possible in the presence of the membrane, and processes similar from the point of view of the physical mechanism can be observed in the course of virus-mediated membrane fusion.

**Figure 1:**
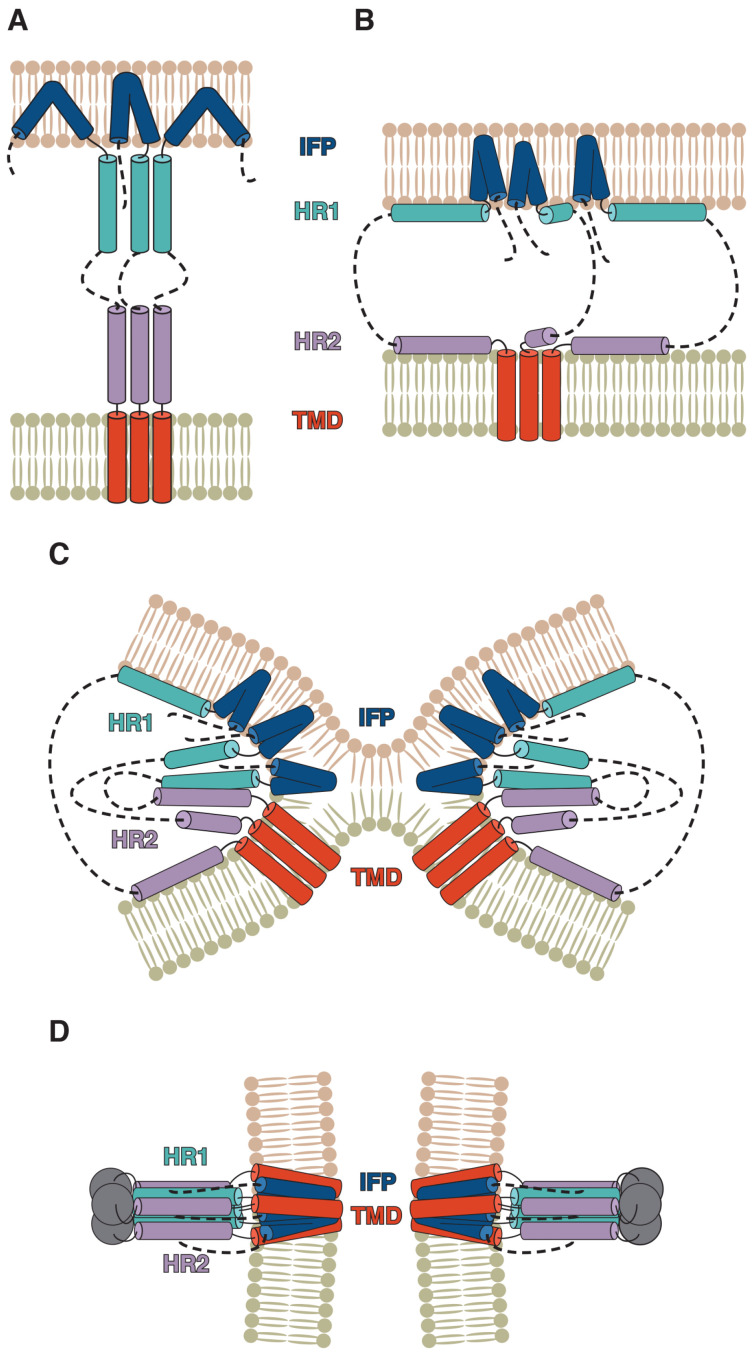
Putative coronavirus spike-mediated fusion mechanism. After the dissociation of the S1 subunit, the internal fusion peptide (IFP) is believed to penetrate the target membrane (**A**). Meanwhile, HR1 and HR2, due to their amphipathic nature, bind to the target membrane and viral envelope, respectively (**B**). As refolding takes place, HR1 and HR2 form hairpin-like structures with each other, resulting in the membranes approaching each other and merging (**C**,**D**), while the protein transitions to the post-fusion state. (Adapted from [12] taking into account the recent results obtained by Shi et al. [10]).

In enveloped viruses, a lipid membrane surrounding the viral capsid aids it in crossing target cellular membranes that operate as hydrophobic barriers. The envelope is also where the fusion proteins produced by the virus are anchored. While the greater portion of the fusion protein is exposed on the virion surface, its TMD ensures the correct positioning of the entire protein. This organisational principle is true for the coronaviral fusion protein known as the spike (S) protein. Spikes are homotrimeric class I viral fusion proteins, as was explicitly demonstrated for the murine hepatitis virus [9], and indeed possess major structural and functional features characteristic thereof, most notably hydrophobic fusion peptides and two heptad-repeat-rich regions responsible for the fusion itself, known as HR1 and HR2. These fragments are at the core of the spike’s complex rearrangement, as the protein folds in two in a jack-knife-like manner, with HR1 forming an antiparallel hairpin structure with HR2 (a trimer of hairpins is formed at the level of the entire protein), while the target membrane captured by the fusion peptide is brought into close proximity with the viral envelope, where, in turn, the spike’s TMD is embedded (see Figure 1). As a result, the virus infects the target cell, while the TMD (or the juxtamembrane (JM) region upstream thereof), following the transition from the pre-fusion to the post-fusion state, is believed to shift from a homotrimeric state to associate with one of the spike’s fusion peptides. A recent cryo-EM structure of the spike in the post-fusion state visualised by Shi et al. [10] in lipid nanodiscs reveals that the main fusion peptide driving this process is likely to be the internal fusion peptide, which eventually forms an α-helical hairpin in the fused membranes, resulting, on the trimer level, in a six-helix membrane-embedded core surrounded by three TMD helices, with which it interacts.

In the pre-fusion and post-fusion states, as well as during all the structural and dynamic perturbations in the course of membrane fusion, the TMD remains inalienable from its immediate environment furnished by the viral envelope, since, like in other membrane proteins, it only exists if surrounded by lipids and cannot be regarded separately from the membrane. For membrane proteins, the lipids are what drives transmembrane (TM) helix association and what predetermines TMD helix–helix interfaces on equal terms with the physicochemical properties of the surface of the helix itself (as extensively reviewed in [11]). Viral fusion proteins are unlikely to be an exception to the rule. In the spike’s case, it would be anchored in a membrane similar in lipid composition to the endoplasmic reticulum Golgi intermediate compartment, as this is where virions bud. At this moment, however, we lack thorough understanding of various stages of membrane fusion effectuated by class I viral fusogens, and only putative schemes and models of this process have been proposed on the basis of fragmentary experimental data (e.g., [12]). Nonetheless, we currently possess information on the spike’s structure at the two extremums of its functionally meaningful refolding, namely its pre-fusion and post-fusion states, although only the latter includes experimentally obtained structural data on the TMD and its localisation in relation to the rest of the protein. In order to gain an exhaustive understanding of this process one would require insights into the behaviour of the TMD, other membrane-proximal protein regions, and the lipids in their vicinity at all stages, also taking into account allosteric effects across the protein, as described for SARS-CoV-2 spike [13], which are likely to affect the TMD.

Structural studies of membrane proteins, be that using solution or solid-state NMR spectroscopy, single-particle cryo-electron microscopy, atomic force microscopy or X-ray crystallography, are challenging insofar as one would require the TM segment both at an adequate concentration and in its native state, i.e., in a hydrophobic environment mimicking the membrane. To date, various approaches have been used to accomplish this task under experimental conditions, including solubilisation in detergent micelles, crystallisation in the liquid cubic phase, reconstitution in bicelles (composed of both detergent and lipid molecules), nanodiscs and liposomes, etc. The choice of the membrane-mimicking environment must take into account the structural method employed (see [14] for a review). Complementary to structural methods are computational tools, such as atomistic and coarse-grained molecular dynamics (MD) and Monte Carlo (MC) simulations. In computational experiments, it is possible to model a lipid bilayer of any composition (e.g., a common approximation of a mostly phospholipid bilayer is zwitter-ionic 1-palmitoyl-2-oleoyl-sn-3-glycerophospholcholine (POPC) along with other well-studied single-component and mixed lipid bilayers). Clearly, this can only be properly performed if the properties of the membrane obtained in the calculations are carefully calibrated taking into account experimental data. Such in silico methods can be used for the prediction of helix positioning relative to the membrane, helix–helix interface makeup and the stability of oligomers of α-helices held together by given inter- and intramolecular contacts. When compared against structural data, computational tools offer valuable additional insights into TMD structure and functioning.

The present review attempts to bring together the existing data on the structural organisation and dynamic behaviour of coronavirus spike proteins’ TMDs and regions adjacent thereto, which are potentially capable of binding to the viral envelope in a peripheral manner. Based on the presented data, criteria that should be met by in silico models of trimeric TMDs of viral fusion proteins homologous to the SARS-CoV-2 spike are identified. The review is structured in such a way as to successively describe individual parts of the TMD, namely the N-terminal aromatic-rich segment, the hydrophobic region and the cysteine-rich (Cys-rich) cluster harbouring palmitoyl modifications, as well as to draw conclusions regarding their behaviour as parts of the entity that is the spike protein.

## 2. Spike TMD Boundaries as Exemplified by SARS-CoV-2 S Protein

Different SARS-CoV-2 spike TMD boundaries were predicted with recourse to TMSEG [15] (TMD residues: 1214-1238) and TMPRED [16] (1216–1235) or proposed on the basis of data from UniProt [17] entry P0DTC2 (1214–1234), as well as in the work by Xia et al. [18] (1213–1237) and Cai et al. [19] (1212-1234). Finally, region 1212–1234 was predicted by Aliper et al. [20] based on the results of an MC conformational search in the presence of an implicit membrane known as a “hydrophobic slab”. In this case, the TMD is flanked by Lys1211 and Cys1235, the first Cys residue among the many to follow downstream.

This correlates well with certain experimental data: one could consider the most C-terminal charged highly conserved lysine residue upstream of the distinctly hydrophobic area, evidently not part of the membrane anchor. Lys and Arg residues have been shown to flank TMDs in some proteins (reviewed in [11]). Downstream of this position, a small region rich in aromatic residues is located, followed by a longer hydrophobic segment and a region rich in Cys residues, whereof even the most N-terminal part has been hypothesised to be membrane-proximal, but not part of the actual membrane-spanning portion of spike [21]. Indeed, in the recent solution NMR study of a peptide corresponding to the SARS-CoV-2 TMD residues 1235 and 1236 (replaced with serines to avoid disulfide bridge formation) were located at the very cytoplasm-facing end of the TM helix [22]. On the other hand, in the recently published cryo-EM structure of the SARS-CoV-2 spike in the post-fusion state [10] C1235 and C1236 were not part of the TM helix. In this structure, the TM helix started at Y1215 and ended at L1234; however, W1212 was also entirely buried in the lipid bilayer, suggesting certain structural continuity across residues 1212 to 1234 (cf. predictions in [20]). In any event, seeing precise boundaries between domains might be fuzzy and might vary between different conformational states. Based on the above data, one would not be wrong considering the first two cysteines as the point where the TM helix ends and the Cys-rich region begins. Cysteine residues have been classified into “clusters”, relatively separate groups interspersed by non-cysteine amino acids.

## 3. Spike TMD Conformation

Whilst a plethora of spike ectodomain structures from various viruses are available in the PDB database, very little structural information has been obtained on the spike’s membrane-anchored region. Structural information available on other class I fusion proteins evidentiates that their TMDs are trimers of α-helices [23,24]. Recently, a trimerisation study was conducted [25] on a peptide corresponding to SARS-CoV-2’s spike residues 1209–1237, in which Met and Cys residues were substituted for Leu and Ser, respectively, amounting to a total of four point mutations compared with the wild-type protein. In dimyristoylphosphatidylcholine/1,2-Dihexanoyl-sn-Glycero-3-Phosphocholine (DMPC/DH_6_PC) bicelles, this peptide assumed a trimeric structure with an Leu/Ile-zipper-like interface. The portion of the peptide whereof the structure was resolved and which spanned the bicelle membrane was mapped as residues 1218 to 1234, corresponding to a rather short TMD fragment 16 residues long (PDB ID 7LC8), while the aromatic-rich fragment was described as unordered. Thus, the propensity of spike protein TMDs to exist in the α-helical conformation has been confirmed, much in line with the general notion that hydrogen bonds formed by polar moieties of the backbone and stabilising the α-helical structure become highly thermodynamically favourable in a hydrophobic environment [26,27]. It is not clear at this time what state of the spike protein this NMR structure corresponds to and at what stage of membrane fusion residues constituting helix–helix interfaces in this study manifest themselves in the actual virus particle. Indeed, sometimes altering the membrane-mimicking environment composition can result in a different functional state of the protein of interest [14,28].

**Figure 2:**
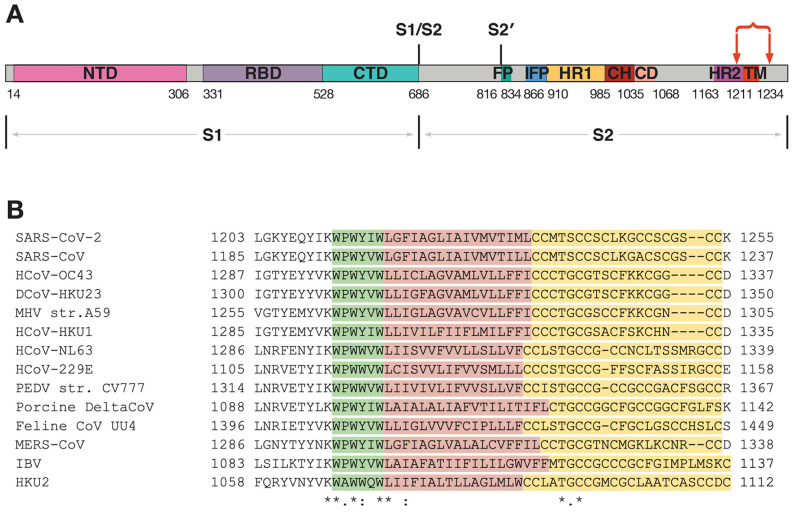
Coronavirus spike proteins and their TMD sequences. (**A**) Spike protein organisation as exemplified by the spike of SARS-CoV-2. The S1 subunit mainly responsible for target cell recognition comprises the N-terminal (NTD), receptor-binding (RBD) and C-terminal (CTD) domains. Downstream of the S1/S2 cleavage site is the S2 subunit effectuating mostly mechanistic membrane fusion; S2 is additionally cleaved at the S2’ site, immediately followed by the fusion peptide (FP), apart from which S2 includes the internal fusion peptide (IFP), HR1, central helix (CH), connector (CD), HR2 and transmembrane (TM) domains. The region of interest within the scope of our review is located between two red arrows. (Adapted from [19]). (**B**) Sequence alignment of the fragment from different coronaviruses indicated by arrows in panel (**A**). The aromatic-rich region, the hydrophobic region and the cysteine-rich region are highlighted in green, red and yellow, respectively. Identical, conserved and semi-conserved residues are designated with the asterisk (*), colon (:) and dot (.) symbols, respectively.

Furthermore, the portion of the SARS-CoV-2 spike upstream of residue 1218 is likely to be conformationally mutable as the protein changes from one functional state to another, which is often the case with JM regions (e.g., [6]). In fact, in an earlier NMR study of SARS-CoV’s spike’s JM region downstream of HR2 (highly homologous to SARS-CoV-2 and highly conserved among coronaviruses in general) in a solution containing dodecylphosphocholine (DPC) micelles, the peptide examined (residues 1185–1202, see Figure 2B) demonstrated a tendency to exist in a helix–loop–helix configuration, with the loop corresponding to the area around K1193 [29]. Circular dichroism spectroscopy [30] only furnished limited evidence of the secondary structure of the peptides corresponding to various parts of the pre-transmembrane region, be that in water or in the presence of lipid vesicles, and the authors predicted a partially ordered structure. Infrared spectroscopy revealed that the pre-TMD from SARS-CoV’s spike (residues 1185–1202) was more likely to assume the α-helical or random coil structure in the presence of DMPC/dimyristoylphosphatidylglycerol (DMPG) liposomes compared with D_2_O solution [31]. Much in the same vein, Liao et al. [32] demonstrated that a peptide corresponding to SARS-CoV spike residues 1191–1200 placed in an aqueous environment transitioned from a β-sheet-like conformation towards a more helical one upon the addition of trifluoroethanol (TFE), a lipid mimetic. W1194 appears to be central to the peptide’s conformational plasticity: once it was substituted with alanine, the peptide tended to be “locked” in the helical state at a broader range of TFE concentrations. In a more recent solution NMR study in DPC micelles [22], a SARS-CoV-2 spike fragment comprising the C-terminal portion of HR2, the TMD and the N-terminal Cys-rich region revealed a tendency for HR2 and the TMD to exist in the helical conformation with a linker loop corresponding to residues 1210 to 1214. It remains to be verified at what stages during fusion the aromatic-rich region assumes a secondary structure enabling it to destabilise the viral envelope and whether this helix downstream of the lysine, when present, might be one with the TM helix itself, even though it might be tricky to pinpoint a strict boundary between the JM region and TMD. Tomographic data corroborated by MD simulations suggest that a hinge area is present between HR2 and the TMD in the pre-fusion spike [33]. In the cryo-EM structure of the post-fusion SARS-CoV-2 spike [10], the boundary between the TMD and HR2 becomes less apparent, as the C-terminal portion of the latter assumes an unordered conformation. The TMD exists as an α-helix from W1217 downstream; its N-terminal aromatic-rich fragment also becomes unordered.

When structural data are not available, one can have recourse to various prediction approaches to furnish TMD models that will be as realistic as possible. To create such models one must first and foremost take into account the known principles of TM helix packing. More specifically, tucked towards the helix–helix interfaces would be the least hydrophobic residues [34,35] with the more hydrophobic side chains facing the lipids of the membrane. Furthermore, helix–helix interfaces would be more likely to feature residues conserved across a range of homologous proteins [34,36]. Obviously, these criteria are not absolute and only reflect a general tendency. However, the structural organisation of a particular TMD is governed by a complex interplay of factors, and the ones cited are considered among the key forces. Furthermore, of note is the positioning of proline residues, prone to form convex-like protrusions on the helix surface and likely to favour the interface rather than the surface exposed to the lipids [35]. Given the position of Pro in the aromatic-rich domain, it might be worth exploring how it fits with the hypothetical conformational flexibility of the N-terminal portion of the TMD and whether it is in any way involved in the conformational rearrangements of the membrane-proximal regions of the spike, be that via switching between the cis- and trans-configurations or otherwise. Importantly, a proline is found in this position across all known geni of coronaviruses except for a very distinct sublineage of coronaviruses (which includes HKU2, see Figure 2B), whereof the spikes possess a lower than 28% identity to their counterparts in other species and which instead have an alanine residue in this position [37]. Various in silico tools could be put to use to predict the subtler aspects of the apparent importance of this position and its role during fusion, e.g., how it is involved in stabilising the trimer, if at all.

## 4. The Aromatic-Rich Region

A small fragment (∼12 residues) rich in aromatic residues, sometimes called the pre-transmembrane region (PTM) is located between the HR2 region, a canonical water-soluble coiled coil involved in membrane fusion, and the hydrophobic TM region (Figure 2). Sainz et al. [30] studied the behaviour of peptides with the sequences of such fragments from the spikes of SARS-CoV (residues 1187–1199), OC43 (residues 1289–1301) and murine hepatitis virus (MHV) (residues 1257–1269, A59 numbering). These peptides showed a propensity for partitioning into large unilamellar vesicles of different compositions and for permeabilising them. In the same vein [38], two peptides corresponding to different SARS-CoV spike fragments downstream of HR2, residues 1185–1202 and residues 1193–1210, demonstrated a high propensity for permeabilising phosphatidylcholine (PC)/cholesterol (Chol)/sphingomyelin (SM) liposomes (a variety of component ratios was tested), the former slightly more than the latter, yet it is interesting that the more C-terminal portion of the peptide, immediately adjacent to the hydrophobic TM segment bordering on a lysine N-terminal (residues 1194–1199), possesses the specified qualities independently of the residues located further upstream. In a follow-up study [31], a PTM-based peptide (residues 1185–1202) demonstrated a distinct affinity to water–lipid interfaces in the presence of liposomes varying in lipid composition; the peptide tended to stay on the interface as opposed to further penetrating the membrane. (The same peptide has since been shown to favour water–lipid interfaces in DPC micelles [29]). In agreement with the data obtained previously, the peptide caused inner monolayer lipid mixing in liposomes, especially phospholipid ones containing phosphatidylinositol and phosphatidylserine, suggesting negatively charged lipids might play a part during viral fusion. In line with this, insertion of the same peptide into membranes was enhanced when liposomes contained negatively charged phosphatidylglycerol [39]. When liposomes contained SM and Chol, thus becoming more similar in composition to lipid raft structures, the peptide was able to permeabilise them with great success [31], but demonstrated a poorer estimated level of insertion [39]. It is noteworthy, though, that the membrane dipole potential (one of the parameters relied on to describe bilayer perturbation) was markedly affected by the presence of this peptide for all liposome compositions studied [39]. Interestingly, the tryptophan residues’ accessibility to the solvent decreased compared with the peptide in an aqueous solution, indicating they were prone to localise in a hydrophobic environment [31,39]. In a solution NMR study of a SARS-CoV-2 spike monomeric fragment spanning lower HR2 to the N-terminal Cys-rich region [22], it was observed that the side chains of W1214 and W1217 are buried in the lipid medium, whilst W1212 tends to be exposed to the solvent. The behaviour of the aromatic cluster as part of the TMD region 1212–1234 was also evaluated in silico using MC simulations with a hydrophobic slab [20]. It was shown that in the most energetically favourable MC-states the entire TM peptide is embedded into the hydrophobic medium and does not expose the N-terminal fragment W1212–W1217 at the lipid/water interface.

The aromatic residues located downstream of the K1193 in SARS-CoV spike have been shown to be of critical importance to viral fusion. Replacement of the tryptophan residues with phenylalanine resulted in a decreased level of virus–cell fusion of pseudoparticles carrying the SARS-CoV spike; even substituting one out of three was sufficient to result in a residual level of entry, while mutants in which two or three tryptophans were thus replaced performed even worse [40]. Similarly, single or double substitutions of the aromatic residues in the PTM (tyrosine or tryptophan) with alanine led to noticeable drops in the levels of cell–cell fusion, as well as of virus–cell fusion for pseudotyped viruses expressing SARS-CoV spike [41], while alanine scanning of the KWPWYIW motif also revealed that these aromatic residues are required for fusion, and phenylalanines in these positions failed to restore fusogenic activity [42]. Intriguingly, the aromatic-rich domain has been shown to possess the capacity to form oligomers stabilised in the presence of glutaraldehyde when a peptide corresponding to SARS-CoV spike residues 1187 to 1202 was studied; however, when the Trp residues were replaced with Ala, this effect was no longer observed [32]. If oligomerisation of this fragment is also the case for the live virus, Trp residues would further contribute to the scope of conformational plasticity the aromatic-rich domain possesses, switching between the monomeric and trimerised states, a transition experimentally shown to be crucial for other class I viral fusion protein TMDs (see [43] for more detail). Liao et al. [32] also demonstrated that a peptide with the sequence of the aromatic-rich region of the TMD was capable of efficaciously binding to the internal fusion peptide (IFP), another membrane-active fragment in the spike (for more detail thereon, see [5]). This observation appears to fully align with the recently published structure [10] of SARS-CoV-2 spike in the post-fusion state obtained in lipid nanodiscs and inclusive of the TMD and the cytosolic part. In this structure, TMD residues in the α-helical conformation are ones from W1217 downstream, while the remainder of the aromatic-rich region assumes a turn- or loop-like shape with P1213 possibly crucial to its formation. Both W1212 and W1217 along with F1220 contribute to the interface between the TMD and IFP, while W1214 and Y1215 face the lipid environment. It remains to be studied at what moment during fusion and in what conformational state the aromatic-rich region’s ability to affect the membrane comes into play.

## 5. The Hydrophobic Region

This segment seems to be important to the spike’s functioning as more than a membrane anchor performing a purely mechanistic role. On the contrary, its amino acid sequence appears to be important to viral infectivity. As Broer et al. [44] demonstrated, chimaeric SARS-CoV spike proteins in which only the cytoplasmic domain (downstream of residue 1234) was substituted with that from vesicular stomatitis virus (VSV) G proteins, on the one hand, and in which both the TMD and endodomain (the entire area downstream of residue 1194) were substituted with corresponding domains of the same G protein, behaved completely differently from each other. SARS-CoV pseudotyped particles (pp) containing the former chimaera retained infectivity to a sufficient extent, whilst pp with the latter were less than 5% as infectious as ones based on the wild-type spike. When the TMD from murine hepatitis virus, a betacoronavirus like SARS-CoV, was substituted for the SARS-CoV spike TMD, such pp were infectious, indicating that the amino acid sequence of this area contributes to processes related to the functioning of the virus as a whole. A similar tendency was observed in the case of cell–cell fusion. In addition to this, VSV-based chimaeric spike trimers were also less stable at a high temperature, which suggests that the wild-type sequence contributes to the stabilisation of the entire protein in a specific way. Interestingly, the area regarded as the TMD in this study contained a total of 34 residues, including the N-terminal aromatic-rich area, the hydrophobic area and some of the C-terminal Cys-rich region. Similar VSV chimaeras were created for MHV, in which the protein segment starting at the first Cys in the Cys-rich fragment was swapped for a sequence from VSV. This chimaera was distinctly non-fusogenic, but once the CCCTGCGSCCF motif was inserted into its original position, fusogenicity was restored [45]. These studies did not explore the role in membrane fusion of the “central” hydrophobic fragment and the N-terminal part of the Cys-rich fragment separately from each other. However, Chang et al. [46] did create an MHV spike mutant that carried the entire native Cys-rich domain, whereas its TM hydrophobic portion was replaced with the TM anchor of glycoprotein D of herpes simplex virus type 1. This chimaera did not display fusion activity, indicating that the sequence of the hydrophobic region of the TMD is crucial to the spike’s functionality, probably manifesting itself at the level of the 3D structure in such a manner as to act in synergy with other segments. Similar data were obtained in a study centering on MHV strain A59 [47]: once the hydrophobic portion was replaced with that from PDGFR, while the Cys-rich segment and endodomain were left untouched, the resulting virus, despite apparently successful incorporation of the spike, failed to be viable, indicating fusion competence might be affected. In a somewhat similar vein, it has been shown that the TMD of MHV could be replaced with that from another coronavirus spike, that of feline coronavirus, and this did not significantly impact the assembly of coronavirus-like particles [48].

An extensive mutagenesis study performed on SARS-CoV spike [40] highlights the importance of specific residues within the TMD to the process of viral fusion. For instance, when two out of three tryptophan residues in the aromatic-rich cluster were substituted with phenylalanines, this resulted in a lack of viral entry for SARS pp, while replacement of only one of these Trp residues resulted in a residual level of entry, indicating that the spike is sensitive to modifications as mild as these. In a similar vein, it was shown how an array of substitutions and insertions (Lys or Ala) affecting the hydrophobic segment were crucial to the adequate functioning of the spike. In an earlier study on MHV strain A59, it was demonstrated that the substitution of I1296 with a Lys residue resulted in a loss of capacity for cell-to-cell fusion, suggesting a charged residue is not tolerated at this last position in the hydrophobic segment immediately preceding the first cysteine cluster [45].

Certain controversy surrounds two amino acid positions in particular, two glycines flanking a GxxxG motif (a sequence commonly known as the glycophorin-like motif) found in some betacoronaviruses. One study conducted for SARS-CoV spike revealed that this motif might be crucial to the trimerisation of the TMD and possibly the entire spike protein, as their substitution with isoleucine resulted in a lower level of trimerisation [49]. Shortly afterwards, another study was published [50], in which mutations of the same residues were addressed (this time G ⇒ L replacements were introduced), but no impact on spike trimerisation was detected. On the other hand, G1205 was found to be crucial to pp infectivity. Single small residues such as glycine and alanine and more complex motifs featuring them within the TMD have proven to be important to the proper functioning of other class I viral fusion proteins (see [43] for a review). In a more recent study, SARS-CoV TMD was demonstrated to be capable of trimerisation in vitro using sedimentation equilibrium analytical ultracentrifugation, while the monomeric, trimeric and higher-order-oligomeric states were speculated to coexist [51]. Similarly, a chimaera was constructed consisting of the spike TMD and RBD located immediately upstream [52], and the TMD turned out to be sufficient for such a chimaera to exist as a trimer, as well as to be recognised by antibodies to the RBD in its ‘natural’ state within the spike, indicating that it was correctly folded. If anything, all of the data cited demonstrate that the spike is clearly extremely sensitive to the precise sequence of the TMD, and its modifications have detrimental consequences, presumably due to the disruption of its optimal packing.

**Figure 3:**
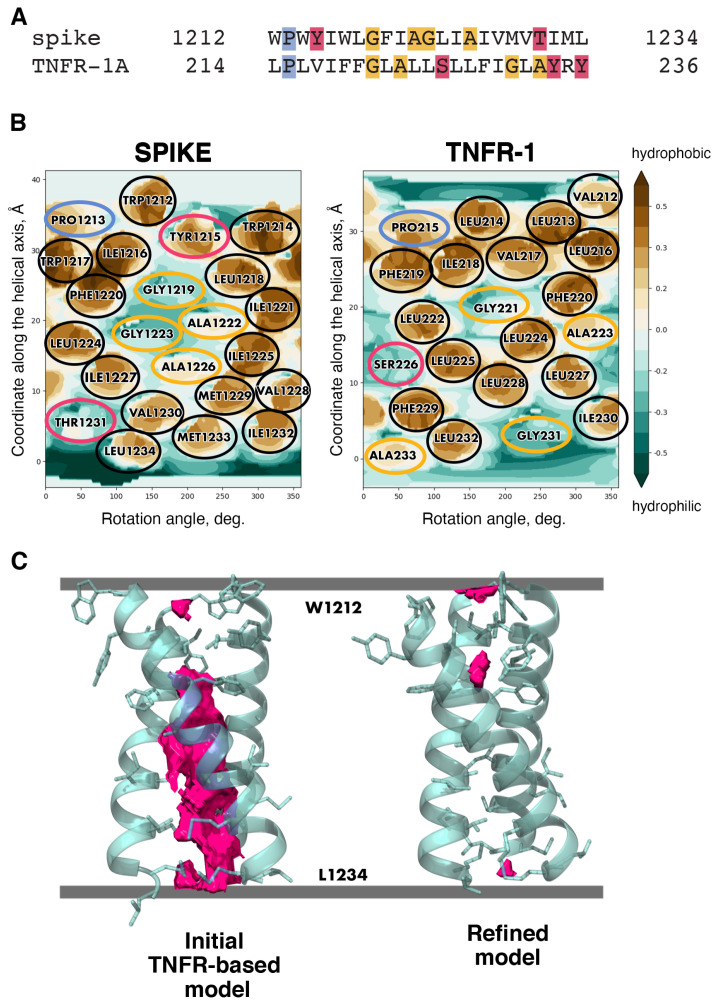
Template-based modelling as applied to the spike TMD. (**A**) TMD sequence alignment for the spike TMD and one of the candidate templates, tumour growth factor receptor 1A (TNFR-1A). Hydrophobic residues (as well as the charged R) are unhighlighted, while proline, small and polar residues are highlighted in blue, yellow and pink, respectively. (**B**) Molecular hydrophobicity potential (MHP) distribution maps for an ideal helix corresponding to S-protein residues 1212–1234 and the TMD monomers of candidate template TNFR-1. Cylindrical projection of the surface MHP distribution is used. Axis values correspond to the rotation angle around the helical axis and the distance along the latter, respectively. An MHP scale (in logP octanol-1/water units) is presented on the right. The maps are coloured in accordance with the MHP values [57], from teal (hydrophilic areas) to brown (hydrophobic ones). Projections of proline, small, polar and hydrophobic residues are encircled in blue, yellow, pink and black, respectively. (**C**) Free volume in the trimer lumen in the initial state built via template-based modelling and in the final trimer refined in the course of MD simulations. Protein chains are partially transparent and are shown in cartoon representation; residues within each chain facing either of the remaining two chains are shown in stick representation. Free volume is rendered as solid pink blocks; membrane boundaries are shown as grey lines.

**Figure 4:**
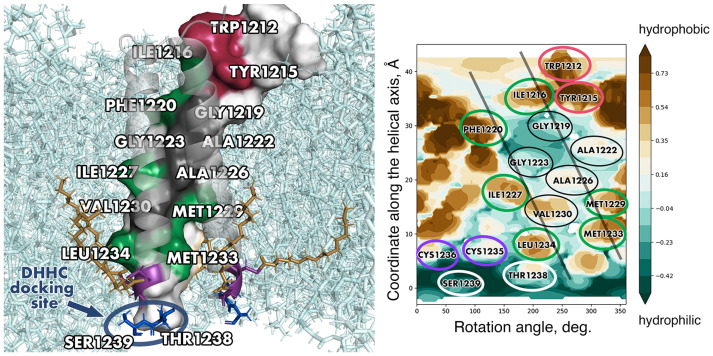
A uniquely stable model of spike TMD built by Aliper et al. [20]. (**Left**) The model in 3D. One of the helices is shown in surface representation; the other two helices are shown in cartoon representation. Identical, conserved and non-conserved residues on the helix/helix interface are coloured dark red, green and dark grey, respectively. Palmitoylation sites, palmitoyl tails and lipids are shown in stick representation and coloured purple, dark golden and cyan, respectively. Residues constituting the hypothesised docking site for the enzyme conducting palmitoylation (DHHC) are coloured dark blue, encircled in dark blue and labelled accordingly. (**Right**) Helix/helix interfaces in one of the chains visualised on a surface MHP map. Identical positions are encircled in red, conserved and semi-conserved residues are encircled in green, and non-conserved residues present on the helix/helix interface are encircled in black. Cys residues and residues constituting the putative docking site for DHHC are encirced in purple and white, respectively. Projections of the other two helices are shown as semi-transparent black lines. For other details, see legend to Figure 3.

Apart from models of the SARS-CoV-2 spike TMD built using diverse automated tools (e.g., [53,54]), a number of others have been created via template-based modelling, which is commonly employed to accomplish such tasks. One of such models [55] was based on a water-soluble coiled-coil fragment; therefore the most hydrophobic surfaces turned out to constitute the helix/helix interfaces, in defiance of the TM helix packing principles (see above). Two more models, one by Woo et al. [56] and another by Aliper et al. [20], ended up converging towards very similar helix/helix interfaces despite being built using two entirely different templates, HIV’s gp41 TMD and human tumour necrosis factor receptor 1A TMD, respectively (see Figure 3 for details on the latter). In these models, helix/helix interfaces were constituted by a number of highly conserved residues (F1220, I1227, etc.) and encompassed the least hydrophobic patches made up by small residues, Gly and Ala (see Figure 4). The viability of model helical oligomers can be evaluated in the course of MD simulations in a lipid bilayer via the assessment of various values such as the model’s stability (root-mean-square deviation (RMSD) for the backbone atoms), free volume inside the model lumen (small values being indicative of helical surfaces being close enough to each other to be stabilised by intermolecular interactions) and the number of non-covalent interactions between the lumen-facing residues and lipids per frame (if it does not grow dramatically, the helix/helix interface does not become more accessible, i.e., it is stable). When tested using this approach, the two models conforming to the known TM helix packing principles proved to be sufficiently robust. Models such as these could conceivably be employed to gain insights into various processes unfolding during membrane fusion taking into account parts of the spike beyond the TMD or to predict the role of specific residues in the stabilisation of the helix/helix interfaces and, by extension, of the spike in general.

**Figure 5:**
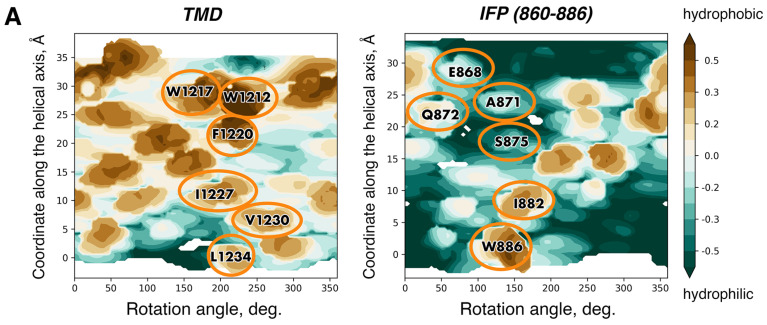

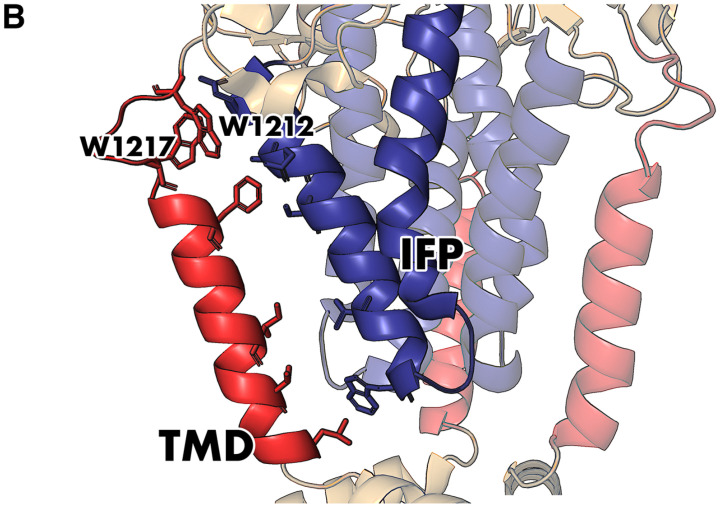
The interface between the internal fusion peptide (IFP) and TMD of the spike in the post-fusion state (structure PDB ID 8FDW [10]). (**A**) Molecular hydrophobicity potential (MHP) distribution maps for the TMD and IFP. See legend to Figure 3 for further detail. Projections of residues on the TMD/IFP interface are encircled in gold. A residue was considered to be on the interface if the area of its solvent-accessible surface went down by at least 25 Å^2^ compared with the monomeric state (for Gly residues, this difference had to be at least 10 Å^2^). (**B**) A 3D structure of the membrane-buried portion of the spike. The TMD and IFP are coloured red and dark blue, while the rest of the protein is coloured beige. The protein is shown in cartoon representation and is semi-transparent apart from one IFP and one TMD interacting with each other, in which residues on the interface are shown in stick representation.

In the post-fusion state [10], the TMD interacts with the IFP, a helical fragment ∼45 residues long that bends double inside the membrane. The resulting structure consists of nine helices, with the inner core formed by the C-terminal halves of the IFP (that continues into HR1 outside the membrane) forming hairpins with its N-terminal portions, which, in turn, interact with the TMD helices positioned peripherally. The IFP’s most hydrophobic residue patches are involved in the stablilisation of the IFP/IFP hairpins, whilst it furnishes less hydrophobic residues to make up the interface with the TMD (Figure 5). The latter, interestingly, contributes W1212, W1217, F1220, I1227, V1230 and L1234, residues that consistently formed helix–helix interfaces in the model of the TMD trimer by Aliper et al. [20] with the exception of W1217, which was on the surface next to the interface and contributed thereto irregularly.

## 6. The Cys-Rich Region

Another element of the spike’s structure vital to its functionality is the cysteine-rich region located downstream of the hydrophobic TM anchor and containing several distinct clusters of cysteines interspersed with other residues. Their importance is not surprising, insofar as membrane-proximal cysteines are potential acylation sites, whereto palmitoyl chains are attached via thioester bonds, which is a common modification in TMDs, including ones from viral fusion proteins [58], that is carried out by enzymes in the zDHHC family [59]. Coronavirus spike proteins have been experimentally observed to be palmitoylated [21,45,60,61], and it has been shown for the spike of SARS-CoV that these post-translational modifications are stable [62]. The Cys-rich fragment has been extensively studied for three betacoronaviruses of particular interest, SARS-CoV [21,62,63], SARS-CoV-2 [60,61,64,65,66] and the prototypical MHV [46,47,67,68,69], as well as one alphacoronavirus, transmissible gastroenteritis virus (TGEV) [70]. Selected experimental data regarding the Cys-rich region obtained in various studies are summarised in Table A1.

The overall tendency appears to be that palmitoylation is important to the spike incorporation into virions, as well as for viral fusion, as shown using virus–cell and cell–cell fusion assays. For different viruses, certain palmitoylation sites have been shown to be more important than others, and synergy-like effects have been observed: palmitoylation at some sites enhances palmitoylation at others. Clearly, the TMD located immediately upstream must be oriented and packed in such a way as not to hinder these processes of utmost vitality to the virus as a whole. It is also important to note that the state of acylation of this domain should, if possible, be taken into account in structural and functional studies of the TMD itself and other membrane-bound S-protein regions. Thus, all-atom MD simulations in a pure POPC bilayer showed a tendency for SARS-CoV-2 TMD, both unpalmitoylated and palmitoylated at C1235 and C1236, to slightly decrease the configurational entropy and/or order parameters of the surrounding lipids compared with pure POPC [20], as is often the case for membrane-spanning proteins. It remains to be further investigated whether the same effect takes place in other model bilayers and whether different lipid compositions might reveal a difference between the palmitoylated and unpalmitoylated versions of the TMD.

Another interesting feature in this part of the spike molecule is a highly conserved threonine residue between cysteine clusters I and II (position 1238 in SARS-CoV-2 spike). It has been shown that this residue alongside the adjacent serine in SARS-CoV-2 is crucial to palmitoylation by DHHC20. One of the possible explanations (as offered upon the analysis of all-atom models of SARS-CoV-2 TMD and DHHC20) is that these two residues constitute a hydrophilic docking site interacting with hydrophilic moieties of the enzyme in the vicinity of the cavity that harbours the substrate [66].

While the Cys-rich region is not part of the TMD per se, not only does it make important contributions to the spike’s functionality during fusion, but it also impacts its lipid environment. Palmitoylation affects the protein’s capacity for partitioning into detergent-resistant membranes (DRMs, in vitro models of dynamic lipid rafts enriched in Chol and glycosphingolipids) [62,68]. A dramatic drop of affinity for DRMs has also been observed for the spike of MHV exposed to 2-bromopalmitate, a palmitoylation inhibitor [69]. As has been remarked previously, the lipid environment is essentially inalienable from the TMD; hence, studying the Cys-rich fragment is bound to consistently reveal facts that are valuable in the context of the TMD’s structure and dynamics.

## 7. Beyond the TMD Trimer

The TMD’s activity during fusion is part of an intricately orchestrated process involving other membrane-active portions of the spike. The fusion peptide or peptides penetrate the target cell membrane, whilst HR1 is believed to align itself parallel thereto and bind to it due to its amphipathic nature, as was experimentally observed via NMR spectroscopy [12]. Meanwhile, a mirror image of the same design probably manifests itself on the viral envelope, with the TMD as anchor and HR2 lying down on the membrane and binding it additionally. It is thought that HR1 and HR2 could thus aid in pulling the two membranes together during spike refolding (see Figure 1). However, under experimental conditions isolated HR2 showed a rather low affinity for the water–lipid interface, inviting the hypothesis that the TMD becomes what the fusion peptide is to HR1, an anchor ensuring it remains sufficiently close to the membrane to bind it. It would therefore be worth trying to verify whether this is true, as well as to examine how HR2 and the TMD might influence each other allosterically, on the level of the monomer as well as on that of the entire trimer. With regard to in silico models of the TMD trimer, their stability could be tested when attached to other parts of the spike trimer to see if the helix/helix interface is robust enough to withstand the presence of the ectodomain or portions thereof. Furthermore, some existing models of the TMD [20,56] both seem to correspond to a specific energy minimum as far as helix packing goes; however, one cannot help wondering whether other energy minima exist and what role helix/helix interfaces existing under these conditions could conceivably play during viral fusion. While we now have access to the 3D cryo-EM structure of the post-fusion spike, including all the membrane-buried fragments, structures of its pre-fusion state inclusive of the TMD have not yet been resolved. Structural data for the isolated TMD obtained via NMR spectroscopy in membrane-mimicking environments (lipid/detergent bicelles) have been published, but such TMDs’ sequences contained amino acid substitutions, which could result in deviations from the wild-type TMD structure. Furthermore, MD simulations in an explicit bilayer revealed that these models are not optimally packed, as they start harbouring noticeable free volume zones [20], which in turn results in trimer destabilisation in a water/lipid environment. As shown by Aliper et al. [20], introducing substitutions to these structures resulting in the wild-type sequence did not generate a tightly packed trimer that was stable throughout MD. It is thus likely that the structural template per se and the corresponding helix/helix interfaces are not optimal for spike TMD organisation. Among models built in silico, one can distinguish two groups: stable and unstable throughout MD simulations, which are tightly and “loosely” packed, respectively. The most stable and tightly packed model of the spike TMD proposed by Aliper et al. [20] was created in an almost “manual mode” by way of iterative adjustment of TM helix packing based on their dynamic “MHP portrait” analysis in the course of MD in a model bilayer. (In this case, one should certainly keep in mind that the exact nature of the membrane-mimicking environment can affect the structural stability of TMDs; the aforementioned simulations were carried out in a POPC bilayer.) This approach had previously been used by the authors to model α-helical TM dimers on the basis of their amino acid sequences (e.g., [71]). However, generalised computational solutions for the reliable prediction of TM trimers’ 3D structures do not yet exist. Detailed analysis of TM helix packing in trimers shall be required to tackle this task, which is of both fundamental and practical importance. Such analyses could be used to design a scoring function for the models generated, much like the PREDDIMER algorithm works for helix/helix complexes [72]. The scoring function, when created, shall allow one to construct models of other class I fusion protein TMDs (from coronaviruses, orthomyxoviruses, etc.), unearth the patterns and peculiarities of their structural and dynamic properties and thus shed light on the subtler details of their functioning in the course of membrane fusion. This, in turn, shall open up prospects for the rational design of compounds (including antibodies, TM “interceptor peptides”, and so on) affecting the TMD and functionally related membrane-active regions in a targeted manner, hindering the optimum performance of the fusion machinery (see [43] for information on class I viral fusion protein TMDs from this perspective, and [8] for a review of diverse non-viral TMDs as potential therapeutic targets allowing one to impact the protein as a whole via its TMD).

It is worth remarking that the highly stable tightly packed model of the spike TMD [20] has already been put to practical use: on its basis the docking site for the enzyme DHHC-acyltransferase 20 (DHHC20), crucial to spike palmitoylation, was predicted and confirmed experimentally via mutagenesis [66]. As TM trimer modelling techniques evolve, the demand for the results of such studies shall grow.

Apart from this, as is evident from the results recently obtained for signal receptors forming TM dimers, upon transition from dimers to higher-order oligomers (e.g., tetramers), synergy effects not previously seen in dimers become detectable using computational methods [73]. It is thus not unlikely that similar phenomena might occur in TM trimers.

In many studies of the spike’s behaviour on a molecular level, its TMD is only regarded as a membrane anchor, without attempting to understand how its structural and dynamic properties affect other membrane-active fragments of the protein such as HR2, HR1, FPs, etc. Meanwhile, it has been elucidated that the spike TMD must possess fundamentally dualistic properties in order to contribute to membrane fusion, which is the main function of the S2 subunit in spike. On the one hand, the TMD must be conformationally rigid at the early stages of this process in order for the aforementioned fragments to dissociate from the initial coiled-coil state and bind to the viral envelope (HR2) and target cell membrane (HR1, FPs). On the other hand, at the subsequent stages the spike TMD must be capable of undergoing significant conformational rearrangement from the pre-fusion trimeric complex into the post-fusion state in which the TMD helices are located peripherally surrounding the IFP core. Conformational transformations of the protein are global in nature and are therefore characterised by high-amplitude movement of large protein fragments. This functionality must be provided at the level of the TMD’s mechanical properties, especially in its N-terminal portion connected to the spike ectodomain and possessing high conformational lability compared with the rest of the protein, taking into account factors like the presence of a proline residue in this segment. Notably, understanding the said molecular mechanisms of viral fusion proteins will require the size of the modelled systems to expand: from the TMD to TMD-HR2, TMD-HR2-preHR2, etc.

Another key influence during fusion, interacting with various portions of the protein, is the membrane. In fact, the TMD is believed to interact both with the viral envelope and the target membrane at some point during fusion, and a variety of mechanisms via which this could take place have been hypothesised (see [74] for more detail). Furthermore, not least due to their peculiar physicochemical properties, tryptophan residues have been shown to contribute to lipid ordering when positioned on the water/membrane interface [75]. In other viral fusion proteins, such as gp41 from HIV, the JM segments containing aromatic residues, even as short as LWYIK, have been shown to be crucial to viral fusion, while N-acetyl-LWYIK-amide has been observed to penetrate deeper into a Chol-enriched lipid bilayer compared with pure phosphatidylcholine membranes, possibly due to aromatic amino acids forming stacking interactions with Chol (reviewed in [76]), thus recruiting it for subsequent membrane rearrangement. Taking into account the structural and functional similarity between the JM regions in retro- and coronaviruses, one could speculate that the latter might effectuate a similar scenario. It has also been demonstrated [60] that palmitoylated spike induces the formation of lipid nanodomains rich in sphingolipids and Chol in its immediate vicinity. While they are not bona fide lipid rafts and the overall lipid composition of the virion is not affected dramatically, these nanodomains clearly perform a specific function during membrane fusion. Cholesterol has been proven to induce high-curvature states in membranes (see [77,78] for a review), which could explain a number of phenomena observed for various class I fusion proteins. For instance, the palmitoylation of haemagglutinin was demonstrated to affect the curvature of the viral envelope, while total Chol content therein remained comparable to that in wild-type influenza virus; the unpalmitoylated phenotype correlated with the formation of fusion pores unfit to transfer the viral genome into the target cell [79]. Cholesterol, via diverse mechanisms stemming from its physicochemical properties, is involved in viral fusion effectuated by class I fusion proteins from orthomyxoviruses, retroviruses, and filoviruses [80,81,82,83]. Finally, it has been discovered that Chol in the viral envelope is required for SARS-CoV-2 to be fusion-competent [84], inviting one to hypothesise that important synergy between the TMD and membrane dynamics might be at play in the case of *Coronavirinae*, which might be worth exploring in greater detail.

It is to be hoped that one could soon arrive at an in-depth understanding of all the subtleties of membrane fusion effectuated by class I viral fusogens if computational, in vitro and structure-based methods like NMR and single-molecule force spectroscopy [85] were used in synergy to reconstruct the complete picture and elucidate which surfaces of the helices constituting the spike TMD interact with other membrane-active parts of the spike, at what stages of viral fusion they do so, and how they cooperate with the lipid environment at each of these stages. 

## Abbreviations

The following abbreviations are used in this manuscript:

Chol	cholesterol
Cys-rich	cysteine-rich
DH_6_PC	1,2-Dihexanoyl-sn-Glycero-3-Phosphocholine
DMPC	dimyristoylphosphatidylcholine
DMGP	dimyristoylphosphatidylglycerol
DPC	dodecylphosphocholine
DRM	detergent-resistant membrane
FP	fusion peptide
IFP	internal fusion peptide
JM	juxtamembrane
MC	Monte Carlo
MD	molecular dynamics
MHV	mouse hepatitis virus
MHP	molecular hydrophobicity potential
PC	phosphatidylcholine
POPC	1-palmitoyl-2-oleoyl-sn-3-glycerophospholcholine
pp	pseudotyped particles
PTM	pre-transmembrane
RBD	receptor-binding domain
SM	sphingomyelin
TFE	trifluoroethanol
TGEV	transmissible gastroenteritis virus
TM	transmembrane
TMD	transmembrane domain
VSV	vesicular stomatitis virus

## Appendix A

**Table A1:** Overview of selected experimental studies conducted on the cysteine-rich domains of selected spike proteins.

Virus	Modifications Introduced to Spike/ Other Factors Present	Results	Ref.
SARS-CoV	First and second cysteine clusters (ALA)	S-mediated cell fusion levels dropped by 55% and 60%, respectively	[21]
	Third and fourth cysteine clusters (ALA)	S-mediated cell fusion levels dropped less markedly	idem
	C1217, 1218A	^3^H-palmitic acid incorporation dropped by 56%	idem
	C1222, 1223, 1225A	^3^H-palmitic acid incorporation dropped by 49%	idem
	C1230, 1232A	^3^H-palmitic acid incorporation similar to wild-type	idem
	C1235,1236A	^3^H-palmitic acid incorporation dropped by 5%	idem
	All 9 Cys residues	The mutant spike was capable of interacting with the M protein, but the mutant virus’s capacity for cell–cell fusion was impaired	[62]
	Fourth cysteine cluster mutated	The mutant failed to incorporate into virus particles. Mutations of any cysteine did not affect interaction with the M protein	[63]
SARS-CoV-2	All 10 cysteines (ALA)	Pseudotyped particles bound to ACE2, but lost the ability to fuse target cells. For virus-like particles, fusion levels dropped 20- to 40-fold	[60]
	Expression in cells where ZDHHC20 and ZDHHC9 were inactivated	Accumulation of the E protein and RdRp dropped by approx. 75%	idem
	C1235, 1236A	^3^H-palmitic acid incorporation dropped by 80%	idem
	C1240, 1241A	^3^H-palmitic acid incorporation dropped by 40%	idem
	Cys clusters III and IV	^3^H-palmitic acid incorporation comparable to wild-type when mutated separately, but dropped more markedly with both III and IV mutated	idem
	All 10 cysteines (ALA)	Background levels of infectivity and cell–cell fusion. Palmitoylation not detected	[64]
	C1235, C1236, C1240, C1241, C1243 (ALA)	Background levels of infectivity and cell–cell fusion. Palmitoylation levels dropped	idem
	C1247, C1248, C1250, C1253, C1254 (ALA)	Infectivity and cell–cell fusion did not alter significantly. Palmitoylation levels dropped less dramatically than for the mutant mentioned above	idem
	All 10 cysteines (ALA)	Palmitoylation levels dropped to zero. Pseudoparticles incorporated wild-type levels of the spike, but the level of pp–cell fusion and cell–cell fusion decreased dramatically	[61]
	2-bromopalmitate exposure	Palmitoylation levels dropped; spike fusion was significantly impaired	idem
	An array of mutants with all possible variants of Cys clusters mutated to Ala or kept intact	Cys clusters I and II proved to be the ones most significantly affecting the palmitoylation of the spike, especially when mutated simultaneously	[65]
	T1238A, S1239A	Both residues are required for palmitoylation	[66]
MHV	A59: C1287, 1288, 1289S	Capacity for cell–cell fusion was lost	[45]
	A59: C1295, 1296S/F1297N	Capacity for cell–cell fusion was impaired	idem
	A59: C1295S/F1297N	Capacity for cell–cell fusion was not affected	idem
	A59: C1287, C1292, C1296, C1304 (each with SER, TYR, TRP)	In all cases, fusogenic activity was markedly impaired, i.e., each position contributes to fusion	[46]
	A59: C1300, C1303, C1304 (ALA)	Single (1304), double (1303/1304) and triple mutant pseudovirions showed fusion levels 2, 20 and 40 times lower compared with the wild-type, respectively. Affinity to CEACAM was not affected. S2 refolding was shown to slow down significantly. Mutations correlated with failure to associate with the M protein	[67]
	A59: deletion of T1290 downstream	Chimaeric protein w/heterologous ectodomain containing the MHV TMD failed to be incorporated into virions, the virus was not viable	[47]
	A59: deletion of T1290-N1302	Spike failed to cause fusion, despite the same deletion not significantly affecting the incorporation of a counterpart chimaera with a heterologous ectodomain into virions	idem
	A59: a variety of C/A, C/G, C/S mutants	A minimum of three Cys residues required for viral replication	[68]
	2-bromopalmitate exposure (palmitoyl acyltransferase inhibitor)	Spike accumulated poorly in virions and failed to associate with the M protein. Infectivity was impaired	[69]
	JHM: C1347F, C1348F, C1348S (homologous to C1295/C1296 in A59)	Double mutant C1347F/C1348S did not demonstrate lower level of virion incorporation, but a much lower infectivity. Double mutant C1347S/C1348F showed a 10-fold drop in infectivity. Single mutant C1348S showed wild-type-level infectivity. Palmitoylation at these positions is not crucial to spike incorporation into virions	idem
TGEV	2-bromopalmitate exposure	Affects infectivity	[70]
	A variety of mutants carrying 3 to 10 C/A substitutions	Mutating all 10 Cys residues affected S incorporation into virions. Three cysteines from any cluster were sufficient for spike incorporation. Interaction with the M protein was not affected	idem
